# Caring behavior and associated factors among nurses working in Jimma University specialized hospital, Oromia, Southwest Ethiopia, 2019

**DOI:** 10.1186/s12912-020-0407-2

**Published:** 2020-03-23

**Authors:** Adugna Oluma, Muktar Abadiga

**Affiliations:** School of Nursing and midwifery, Institute of Health Sciences, Wollega University, Nekemte, Ethiopia

**Keywords:** Caring, Caring behavior, Nurses, Jimma, Ethiopia

## Abstract

**Background:**

Nursing care behavior and nurse’s perception of effective care behavior is an act, conduct, and mannerism enacted by professional nurses that convey concern, safety, and attention to the patient. Behavior associated with caring has a paramount role in linking nursing interaction to the client in experiences but, the concept is ambiguous and elusive toward different scholars to reach on common understanding. Only a few studies have been done on the caring behavior and associated factors globally, and no study was done in this study area. Therefore; the purpose of this study was to assess caring behavior and its associated factors among nurses working in Jimma University specialized hospital, southwest Ethiopia.

**Methods:**

An institutional-based cross-sectional study design was conducted on a sample of 224 nurses working in Jimma university specialized hospital from March 20–April 20, 2019. Data were collected by a self-administered questionnaire. Descriptive statistics including frequency table, mean, standard deviation and percentage were employed. Bivariate and multiple linear regression analysis was used with regression coefficient (β), coefficient of the determinant (R^2^), CI 95% and *p* < 0.05 were used for statistical significance.

**Results:**

The overall proportion of nurses caring behavior was 80.3% which was mostly measured in terms of professional –technical (82.9%) and psychosocial (81.3%) dimension. Job satisfaction as personal satisfaction (beta = 1.12, *p* = 0.00), professional satisfaction, (beta = 1.07, p = 0.00), joint participation in caring process (beta = 0.58, p = 0.00,) satisfaction with nurse management (beta = 0.85, *p* = 00) were significantly associated with caring behavior.

**Conclusion:**

The proportion of nurses who had a high perception of caring behavior was found to be lower. Thus, all predictors have their own effect on enhancing job satisfaction, improving and creating conducive management and working environment to increase caring behavior. Further comparative studies involving multidisciplinary and patient point of view were recommended.

## Background

Caring is a principal and unique concept in nursing which is described as human acts of doing something with people, for people, to people and as people. It can be effectively demonstrated and practiced interpersonally that result in the satisfaction of human needs. It represents an attitude of occupation, concern, responsibility and affective involvement with the others. Nursing care behavior and nurse’s perception of care behavior is an act, conduct, and trait enacted by professional nurses that provide concern, protection, and attention to the patient [1].

Conceptually, caring behaviors have two major components. The first component is instrumental behaviors which are associated with technical and physical behaviors. The second components are expressive behaviors that deal with psychosocial and emotional behaviors which include the provision of loyalty, confidence, hope, and emotional kindness for the patients [2].

Caring is universal as well as central in the art and science of nursing practice that includes all aspects of delivering nursing care to patients. Caring is a basic nurses’ attitude and daily life events as a professional and individual which occur when a nurse comes in contact with a client and expressed through actual nursing acts and behaviors. Thus, caring requires the spiritual, moral, personal and social engagement of a nurse with a commitment to self and other community [3].

Nurses have a professional responsibility to give high-quality nursing care for a better patient outcome. All nursing activities are verified through nurses caring behaviors. However, the lack of professional caring of nurses leads to reduced wellbeing and the health of the patients. Therefore, nurses caring behaviors can influence patient satisfaction and perceived quality of nursing care [4, 5]. A study showed that approximately 10–30% of general hospital nurses rated the quality of care on their ward as fair/poor and up to 50% felt that the quality of patient care had deteriorated [6].

There was a study that indicated nurses are more emphasized on the expressive aspects of caring such as listening to the patient and less observable aspects of care like patient monitoring. Complaints of poor attitude among health care workers toward patient care were increasing because of the perception that health care professionals are increasingly giving impersonal care especially in overcrowded settings [7].

The concept of caring and caring behaviors has different perceptions among patients served for various cases. Study showed that patients with cancer problems emphasized more for affective caring actions whereas intensive care patients perceived equally the technical and compassion serves. However, patients in the emergency unit emphasized more to technical aspects. A patient in medical-surgical care deals more to physical caring competency and the practical ability to deliver the general well being [8].

There was a study that describes factors inclined caring behaviors including the technique used for assigning nurses for patients, lack of time and support from other colleagues. It is also known that the frequency of visiting the patients might lead to a higher nursing care provision than others that induces a significant effect on nurses caring behaviors [9].

Studies undertaken among hospitalized patients showed those professional nurses and patients’ perceptions of caring are not similar. Patients focused on the technical aspects of nurses’ work in particular, physically ill patients stress task-orientated behaviors whereas nurses value psycho-social skills. Major dimensions of determinants influencing nurses’ perception of caring behavior were the nurse’s characteristics, educational background, workload, job satisfaction and working place [10–12]. Another study showed factor influencing nurses caring behaviors where the care environment, low staffing and support for nurses in the working environment [13, 14].

Even though caring is an important concept in nursing, it is complex, an intangible concept and difficult to measure. Culture and values affect the understanding of the concept of caring. There is also a paucity of research on specific programmatic efforts to enhance nurses caring behaviors among nurses. Therefore, this study was aimed to assess caring behavior and associated factors among nurses in Jimma University specialized hospital, southwest Ethiopia.

## Methods

### Study setting and population

This study was conducted in Jimma University specialized Hospital from March 20–April 20, 2019. The institutional-based cross-sectional study design was employed on 224 nurses working in Jimma university specialized hospital. All nurses in Jimma University specialized Hospital was the source population and the sampled nurses working in different wards present during the data collection was the study population. All nurses working for more than 6 months in the hospital were included Nurses who were not present during data collection period were excluded from the study. Nurses who were not willing to take part in the study due to inclination and other unknown reason were also excluded from participating in the study.

### Sample size determination and sampling techniques

The sample size of the study was calculated using the formula for estimation of a single population proportion with the assumptions of 95% Confidence Level (CL), marginal error (d) of 0.05. Taking a proportion of 0.682 (68.2%) from the previous study conducted in Gondor [14], and by adding a non-response rate of 10%, a total of 224 nurses were enrolled in the study after using the correction formula. A simple random sampling method was used to select the study participants who were involved in this study.

### Data collection tool and procedures

Data was collected using a structured questionnaire. Data collection tools consist of five-part questionnaires: Demographic related questions, Caring dimension inventory scale adapted from the previous study originally developed by Lea and Watson in 1996 with the reliability of Cronbach alpha 0.90 [15]. The job satisfaction scale is taken from the job satisfaction scale developed by Warr et al. 1979 [16]. Interaction scale is adapted from the nurse-physician collaboration scale developed by Rei Ushiro 2009 [17] which has the reliability of Cronbach alpha of 0.80 [17] and Work Environmental Scale adapted from tools developed by Moos, 1994 [18]. A Close-ended self-administered structured questionnaire was distributed to participants by trained data collectors. Data was collected by 3 BSc nurses and 1 supervisor for the duration of approximately one-month duration.

### Data processing and analysis

The data were cleaned and entered into Epi data version 3.1 and then exported to SPSS window version 20.0 for analysis. Univariate analysis like simple frequencies tables, percentages, mean, standard deviation, bar chart, radar chart and pie chart were used extensively. Bivariate linear regression analysis was used to determine independent predictors on outcome variable with regression coefficient (B). Significance was concerned at *p*-value < 0.05 with 95% confidence interval. Multiple linear regression analysis by the coefficient of the determinant (R2) was used to predict the outcome variable with the backward fitness approach in order to get the final significant predictors.

### Data quality control

Data were cleaned, coded and checked for consistency and completeness. The principal investigator prepared the template and entered data using Epi Data version 3.1. Finally, after missing value and incorrect entry checked the data was exported to SPSS version 20. Five percent (5%) of the questionnaire was pre-tested on nurses at Shenen Gibe hospital. One-day training was also given for data collectors and supervisor.

## Results

### Socio-demographic characteristics of respondents

Two hundred twenty-four participants participated giving a response rate of 97.8%. The majority of the respondents 111(50.7%) were female and with regards to marital status, two thirds 132 (60.3%) were single respondents. Most of the respondents186 (84.9%) had work experience less than 5 years. Majority111 (50.7%) were fluent speakers of Afan Oromo followed by Amharic 92 (42%) language. Concerning their educational status majority 123(56.2%) hold diploma and staff nurses. The study also showed that the majority of 87(39.7%) were working in surgical wards and as well as orthodox Christian 84 (38.4%) followers. (Table 1).

**Table 1:** Socio- demographic characteristics of nurses working in Jimma University specialized Hospital, 2019 (*n* = 224)

Variables	Category	Number	Percent
Sex	Male	108	49.3
	Female	111	50.7
Ethnicity	Oromo	147	67.1
	Amhara	64	29.2
	Others	8	3.7
Marital status	Married	87	39.7
	Single	132	60.3
Educational status	Diploma	123	56.2
	Bachelor degree and above	96	43.8
Language	Afan Oromo	111	50.7
	Amharic	92	42.0
	Others	16	7.3
Age (year)	15–24	175	79.9
	≥34	44	20.1
Work experience (year)	0–5	186	84.9
	≥6	33	15.1
Working unit	Medical ward	59	26.9
	Surgical ward	87	39.7
	Pediatric ward	54	24.7
	Intensive care, psychiatry and maternity	19	8.7
Religion	Orthodox	84	38.4
	Muslim	71	32.4
	Protestant	58	26.5
	Others	6	2.7
Position	Staff nurse	196	89.5
	Nurse leader (manager)	23	10.5

### Level of nurses caring behavior

Caring behavior was measured in terms of a psychosocial, professional-technical, appropriate and inappropriate aspect of caring behavior. In the current study caring behavior was measured in terms of emotional (psychosocial) and affective (technical-professional) dimension. Thus, the mean and average mean score of each component were psychosocial 40.75 ± 8.94 (81.5%) and professional –technical 24.87 ± 5.55 (82.9%). The mean and standard deviation of the overall scale was 100.36 ± 19.24 (80.3%). The level of agreement with caring behavior was measured in terms of low, medium and high through calculating the mean difference of their agreement. So, low70 (32%), medium 79(36.1%).

### Job satisfaction among the respondents

**Fig. 1:**
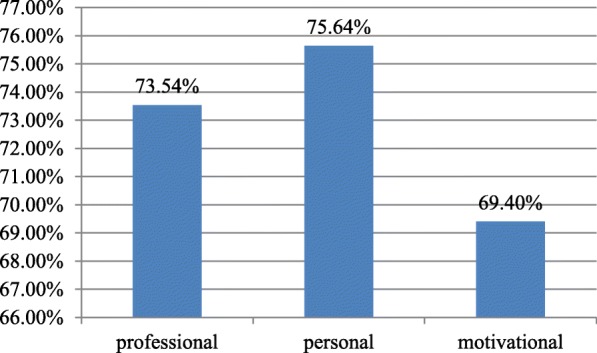
Bar graph illustrating Job satisfaction among nurses working in Jimma University specialized hospital, 2019

The mean and standard deviation of each component of job satisfaction were professional satisfation18.46 ± 5.04 (73.54%), personal satisfaction 18.91 ± 4.53 (75.64%) and satisfaction with motivation and prospect 17.35 ± 4.86 (69.4%) (Fig. 1).

### Interactions (nurses- physicians) related factors

**Fig. 2:**
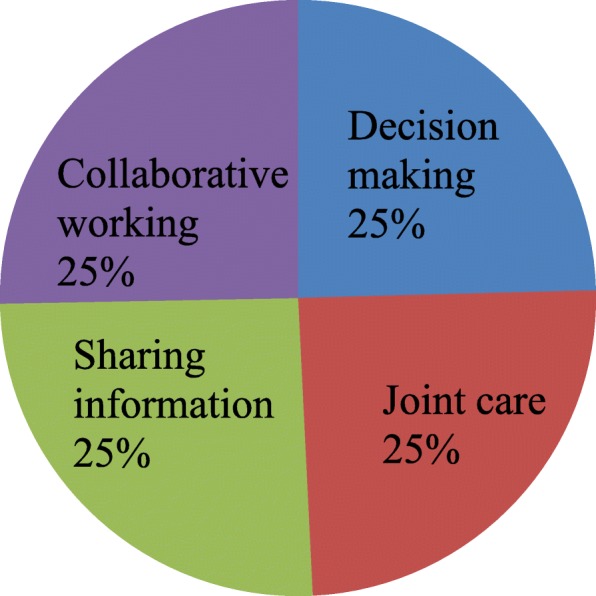
Pie chart illustrating Interaction components among nurses working in Jimma University specialized hospital, 2019

The mean score of interactions related factors with respect to joint participation in decision-making process 24.82 ± 5.85, joint participation in client care 21.30 ± 5.23, sharing patient information 18.37 ± 4.35 and collaborative working 14.59 ± 3.64. The average percentages of the mean of all components were: joint participation in decision-making process70.91%, joint participation in client care 71%, sharing patient information 73.48% and collaborative working 72.95% (Fig. 2).

### Caring environment (organizational factors)

**Fig. 3:**
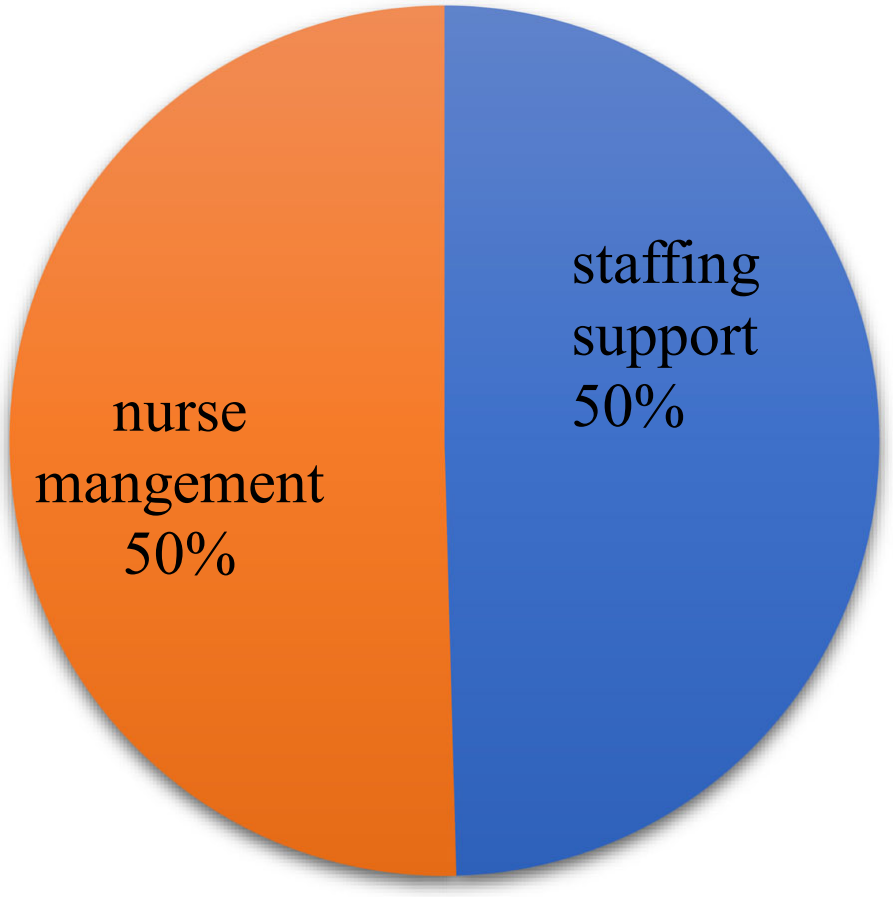
Pie chart illustrating caring environments among nurses working in Jimma University specialized hospital, 2019

Each component has the mean and standard deviation of satisfaction with staffing and support 15.62 ± 6.01 (average mean of 62.48%) and satisfaction with nurse management 19.01 ± 6.99 (average mean of 63.57%) (Fig. 3).

### Bivariate linear regression analysis

Bivariate linear regression analysis revealed significantly associated variables with caring behavior at *p* < 0.05. Among background variables, age, religion (orthodox), working unit (surgical and pediatric wards), professional satisfaction, personal satisfaction, satisfaction with motivation and prospect, joint participation in decision-making process, joint participation in client care, sharing patient information, collaborative working, satisfaction with nurse management, number of patient per shift and plan to leave the hospital were variable significant at bivariate level (Table 2).

**Table 2:** Bivariate linear regression analysis of factors associated with caring behaviour among nurses in Jimma University specialized hospital, 2019 (n = 224)

Variables	Outcome variable: caring behavior		
	Unstandardized B	P- value	CI at 95%
**Background variables**			
Age 15–24 (year)	6.646	0.04	(0.299.12.993)
Age > =25(year)	6.646	0.04	(0.299.12.993)
Religion (orthodox)	6.780	0.010	(1.649,11.911)
Surgical ward	−6.941	0.009	(−12.106, −1.776)
Pediatric ward	7.614	0.011	(1.744,13.484)
**Job satisfaction variables**			
Satisfaction with prospect and motivation	1.278		
Professional satisfaction	1.766	0.00	(1.314,2.219)
Personal satisfaction	1.888	0.00	(1.378,2.397)
**Interaction (interdisciplinary) variables**			
Joint participation in decision making	0.963	0.00	(0.543,1.384)
Joint participation in client care	1.239	0.00	(0.776,1.702)
Sharing patient information	1.196	.0.00	(0.626,1.766)
Collaborative working	1.598	0.00	(0.924,2.272)
**Care environment (organizational) variables**			
satisfaction with nurse management	0.49	0.008	(0.128,0.853)
**Workload and intention to leave variables**			
Number of patients per shift	0.904	0.00	(0.502,1.306)
Plan to leave the hospital	−6.146	0.019	(−11.276, −1.017)

### Multivariate linear regression analysis

Variables significantly associated with caring behavior in the multivariate analysis include religion, being working in a surgical ward, personal satisfaction, professional satisfaction, satisfaction with staffing and support satisfaction with staffing joint participation care process. Hence, a unit increases in personal satisfaction increase caring behavior by an average of 1.12(beta = 1.12, *p* = 0.00, CI at 95%) whereas a unit increase in professional satisfaction increase caring behavior by an average of 1.07(beta = 1.07, CI at 95%). Similarly, a unit increase in joint participation in caring process increase caring behavior by an average of 0.58(beta = 0.58, p = 0.00, CI at 95%) as well with regard to organizational factors, a unit increase satisfaction with nurse management increase caring behavior by an average of 0.85(beta = 0.85, *p* = 00, CI at 95%). Overall the variance by 41% of caring behavior is due to the effect of all predictors as summarized in the final model of the study (R2 = 0.412, *p* = 0.00, F = 16.250). This indicates that variance by average 59% of caring behavior was due to other factors (Table 3).

**Table 3:** Multiple linear regression analysis of factors associated with caring behaviour among nurses in Jimma University specialized hospital, 2019 (n = 224)

Variables	Outcome variables: Caring behaviour		
Background variables	Unstandardized B	P-value	CI at 95%
Work unit (surgical ward)	−6.730	0.002	(−11.025, −2.436)
Work unit (Intensive care, psychiatry and maternity wards)	−7.834	0.054	(−15.447, −0.220)
**Job satisfaction variable**			
Personal satisfaction	1.119	0.00	(0.611,1.627)
Professional satisfaction	1.072	0.00	(0.604,1.539)
**Interaction (interdisciplinary) variable**			
Joint participation in caring process	0.584	0.007	(0.159,1.009)
**Care environment (organization)**			
Satisfaction with nurse management	0.852	0.00	(0.399,1.304)
Satisfaction with staffing and support	−1.140	0.00	(−1.657, −0.623)

Accordingly, the final model of the study:

Caring behavior = 16.25–6.730 (Being working in surgical ward) + 1.12 (Personal satisfaction) + 1.07 (Professional satisfaction) + 0.58 (Joint participation in caring process) + 0.85 (Satisfaction with nurse management) -1.14 (Satisfaction with staffing and support) + 4.35.

## Discussion

The finding of this study showed that the proportion of nurses who had caring behavior was 80.3%. A relatively high proportion of nurses had professional – technical (82.9%) caring behavior compared to psychosocial (81.5%) caring behavior. This indicates that nurses more perceived concrete observable aspects of caring behavior than expressive caring behavior. This finding is similar to study conducted in Gondor and Sweden in which nurses have perceived the technical- professional aspect of caring behavior than psychosocial caring behavior. This similarity might be due to the nature of their profession in which nurses pay special attention mainly involving practical caring rather than motivational concern [14]. However, this finding is in contrast with the study done in Japan and Jordan, in which a high proportion of nurses have perceived a psychosocial (emotional) aspect of caring behavior. This might be because of the difference in organization nature, prevailing attitude given by society [7, 19]. The finding of the study also revealed that nurses’ job satisfaction was associated with caring behavior. Nurses who had personal satisfaction with their job had high caring behavior which is consistent with a study conducted in Gondor on the perception of caring behavior [14, 20].

On the other hand, a cohort study conducted in South Africa revealed that caring behaviors related to job dissatisfaction reflected in increased duration absenteeism [21]. In this study, caring behavior is positively associated with collaborative working as measured joint participation in the client care process among nurses and physicians (B = 0.54, *p* < 0.007, CI95%) which is similar to the study conducted by Lu et al. [22]. However, the study conducted in Slovenian hospital showed those nurses and physician involvements in teamwork were low compared with the current study [23]. These variations might be due to the difference in organizational support, leadership style and the level of salary.

With regard to caring environment, nurses caring behaviors were significantly associated with caring environment as measured presence empowering nursing leader management, staffing and support (B = 0.85, *P* = 0.00) which is consistent with study conducted in New York, Korea, and Saudi Arabia found that nurses who viewed the working environment as empowering were more likely provide high caring behavior [3, 5, 16]. In line with these findings, a study conducted in Australia also indicates a relationship between a nurse’s autonomy and strong ward management with caring behavior [2, 24]. The current study also indicates the presence of inverse relation with working units like intensive care unit, psychiatric and maternity wards which is inconsistent with the study conducted by Ferrous H. Omar [25]. This disparity might be due to presence strictly followed up, unfavorable behavior clients in the psychiatric ward, and the presence of workload leads to a low perception of caring behavior.

### Limitation of the study

The cause and effect relationship cannot be confirmed in this study since the research design is cross-sectional in nature.

## Conclusion

The proportion of nurses who had a high perception of caring behavior was found to be lower. This study found that nurses’ personal and professional satisfaction with their job was positively related to caring behavior. Moreover, nurses caring behavior enhanced with increased empowered nurse’s management in coordinating, planning, controlling the whole process of caring behavior. Furthermore, caring behavior was positively associated with joint participation in client care especially among nurses and physician which is the most pivotal role in the provision of quality care services for the client as a team.

## Data Availability

The data used during this study are available from the corresponding author on reasonable request.

## Abbreviations

CARE-Q: Caring assessment report evaluation questions
CBA: Caring behavior assessment
CBI: Caring behavior inventory
CDI: Caring dimension inventory
DNCB: Determinants of nurse caring behavior
ICU: Intensive care unit
JUSH: Jimma University Specialized Teaching Hospital
OPD: Outpatient department
USA: United State of America
